# Genome-wide analysis of LTR-retrotransposons in oil palm

**DOI:** 10.1186/s12864-015-2023-1

**Published:** 2015-10-15

**Authors:** Thierry Beulé, Mawussé DT Agbessi, Stephane Dussert, Estelle Jaligot, Romain Guyot

**Affiliations:** CIRAD, UMR DIADE (IRD, UM), 34394 Montpellier, France; IRD, UMR DIADE (IRD, UM), 34394 Montpellier, France; IRD, UMR IPME (IRD, CIRAD, UM), 34394 Montpellier, France

**Keywords:** Oil palm, Transposable elements, Retrotransposons, LTR

## Abstract

**Background:**

The oil palm (*Elaeis guineensis* Jacq.) is a major cultivated crop and the world’s largest source of edible vegetable oil. The genus *Elaeis* comprises two species *E. guineensis*, the commercial African oil palm and *E. oleifera*, which is used in oil palm genetic breeding. The recent publication of both the African oil palm genome assembly and the first draft sequence of its Latin American relative now allows us to tackle the challenge of understanding the genome composition, structure and evolution of these palm genomes through the annotation of their repeated sequences.

**Methods:**

In this study, we identified, annotated and compared Transposable Elements (TE) from the African and Latin American oil palms. In a first step, Transposable Element databases were built through *de novo* detection in both genome sequences then the TE content of both genomes was estimated. Then putative full-length retrotransposons with Long Terminal Repeats (LTRs) were further identified in the *E. guineensis* genome for characterization of their structural diversity, copy number and chromosomal distribution. Finally, their relative expression in several tissues was determined through *in silico* analysis of publicly available transcriptome data.

**Results:**

Our results reveal a congruence in the transpositional history of LTR retrotransposons between *E. oleifera* and *E. guineensis,* especially the *Sto-4* family. Also, we have identified and described 583 full-length LTR-retrotransposons in the *Elaeis guineensis* genome. Our work shows that these elements are most likely no longer mobile and that no recent insertion event has occurred. Moreover, the analysis of chromosomal distribution suggests a preferential insertion of *Copia* elements in gene-rich regions, whereas *Gypsy* elements appear to be evenly distributed throughout the genome.

**Conclusions:**

Considering the high proportion of LTR retrotransposon in the oil palm genome, our work will contribute to a greater understanding of their impact on genome organization and evolution. Moreover, the knowledge gained from this study constitutes a valuable resource for both the improvement of genome annotation and the investigation of the evolutionary history of palms.

**Electronic supplementary material:**

The online version of this article (doi:10.1186/s12864-015-2023-1) contains supplementary material, which is available to authorized users.

## Background

Transposable Elements (TEs) are mobile and parasitic nucleic acids that can be distinguished according to their respective mode of transposition. Class I elements or retrotransposons use a RNA intermediate through a “copy and paste” mechanism that may result in an exponential increase of copy number within the genome, whereas Class II elements or transposons are excised from their original genomic insertion (“cut and paste” mechanism) and transpose as DNA molecules [1]. TEs make up a significant fraction of many eukaryotic genomes and, in plants, the increase in TE content is strongly correlated with the increase in genome sizes observed amongst Angiosperms, from 10 % in Arabidopsis [2] to up to 80–85 % in cereals (maize: [3]; wheat: [4]; barley: [5]; see also [6–8] for reviews).

Because of their replication mode, retrotransposons constitute the most abundant TE class. Among them, those with Long Terminal Repeats (LTRs), belonging to *Gypsy* and *Copia* super-families are largely predominant in the genomes of flowering plants [1, 9]. When annotating TE sequences in genomes, further distinction is between autonomous and non-autonomous elements based on the presence or the absence, respectively, of both the POL and GAG coding domains that are required for transposition, regardless of whether these sequences are actually functional [1, 10].

LTR-retrotransposons have significant effects on genome instability through homologous recombination between copies, with potentially long-term consequences on genome evolution [11, 12]. LTR-retrotransposons tend to accumulate within pericentromeric heterochromatin [13] and they also contribute to the formation of functional centromeres in plants [14].

In addition to their effects on gene and genome structure, the insertion of LTR retrotransposons may also affect the regulation of nearby genes and this can in turn result in the emergence of phenotypic variation [15]. Indeed, TEs are targeted by powerful epigenetic repressive mechanisms that ensure their maintenance in a stably silenced statgenome sequences. Phylogenetic trees were based one and these TE-silencing processes share a number of components with pathways that are responsible for the epigenetic regulation of host genes expression [16]. Once an element has been transcriptionally inactivated, it accumulates mutations over time and loses the ability to transpose autonomously. However, even elements that are both transcriptionally silent and immobile can be co-opted by the host genome to provide new beneficial features for both gene regulation and genome evolution through the rewiring of regulatory networks, a phenomenon known as exaptation or molecular domestication [10, 17–20].

The epigenetic repression of TE activity can be transiently alleviated as a result of environmental stresses (heat, cold, UV light, pathogen attack…) and this reactivation, which can affect a variable fraction of the TE populations, is thought to contribute to the short-term response to changing environmental conditions [12, 15, 21–23]. Tissue culture processes, in particular, are well-known triggers of LTR-retrotransposons remobilization [24], as illustrated by the examples of *Tos17* in rice [25], *Tnt1* [26] and *Tto1* [27] in tobacco and *BARE-1* in barley [28]. Ultimately, these reactivated elements can contribute to the phenomenon of somaclonal variation and promote the emergence of altered phenotypes [29–31].

The oil palm (*Elaeis guineensis* Jacq.) is a major cultivated crop and the world’s largest source of edible vegetable oil. The genus *Elaeis* comprises two species: *Elaeis guineensis* Jacq. (Eg) originates from West Africa and *Elaeis oleifera* Cortés (Eo) is found in Central and South America. The inter-fertility between both species allows the use of interspecific hybrid populations in breeding programs.

The African oil palm (*Elaeis guineensis*) is an example of an economically important crop that is commercially propagated through the *in vitro* cloning of high oil-producing individuals. The unpredictable incidence of the *mantled* floral variant among the clonal progeny and its negative impact on oil yields [32] has prompted the search for the molecular mechanisms underlying this phenotype. The reversibility and heterogeneity of the variation have led to the hypothesis of an epigenetic origin, which has been supported by the characterization of a significant DNA hypomethylation of the *mantled* genome [33–35]. Because of the abovementioned well-documented relationship between TE activity and certain somaclonal variations, several attempts have been made to identify some of the LTR retrotransposons of oil palm [36, 37] or to find evidence of their mobilization as a result of the genome-wide hypomethylation found in *mantled* tissues [38]. These studies were however inconclusive at the time, presumably due in part to the lack of genome-wide sequence information allowing only the investigation of individual elements. Therefore a thorough study of LTR retrotransposon populations throughout the oil palm genome and in connection with the *mantled* variation is still warranted. In order to achieve this, it is necessary to first identify and classify TEs from the recently published African oil palm genome assembly [39], as well as from the draft sequence of its Latin American relative (*E. oleifera*) for comparison purposes. To further facilitate data mining, software tools allowing the identification of the structural features of TEs from high-throughput sequencing data have been developed [40–42].

In the present study, we have conducted a genome-wide annotation of Transposable Elements from the publicly available African and American oil palm genome sequences, with a focus on LTR retrotransposons. As a preliminary step, we have built a Transposable Element database and analyzed the TE content for each of the two oil palm genome sequences in order to compare their respective TE populations. We have further identified putative full-length LTR retrotransposons in the *E. guineensis* genome and characterized their structural diversity, chromosomal distribution and estimated their evolution through time. In addition, we have analyzed their transcriptional activity in a variety of organs. Our results provide insights on the LTR retrotransposon landscape and evolution in both the oil palm genomes and constitute a valuable resource for the improvement of their respective genome assembly. Ultimately, the resulting oil palm retrotransposons dataset paves the way for further investigating the role of these elements in the *mantled* somaclonal variation.

## Results

### *De novo* construction and analysis of consensus TE databases

**Fig. 1:**
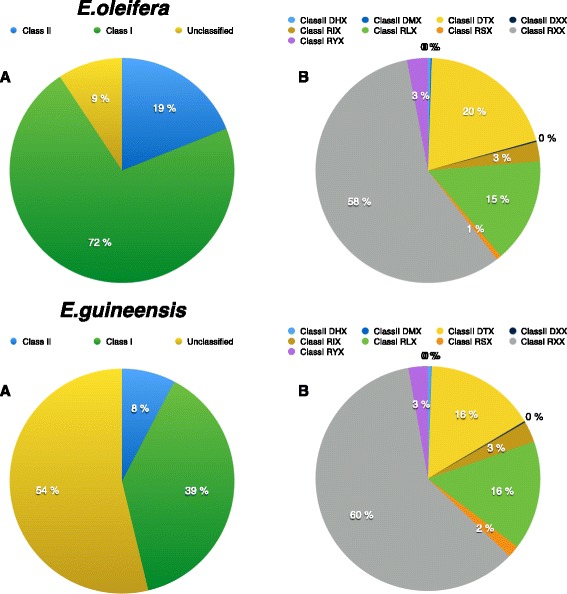
Overview of the TE contents of the *E. oleifera* and *E. guineensis* genome sequences, as identified and classified by the REPET software Top panel *E. oleifera;* bottom panel *E. guineensis.* a: classes; b: orders. Class I elements: RIX LINE; RLX LTR Retrotransposons; RSX SINE; RXX unclassified and Non-autonomous Retrotransposons; RYX DIRS. Class II elements: DHX Helitron; DMX Maverick; DTX TIR Transposons; DXX MITE

A total of 991 *E. oleifera* (Eo) and 846 *E. guineensis* (Eg) scaffolds were used for self-comparison in order to detect repeated sequences within each dataset. After clustering, 4025 and 10,193 consensus repeated sequences were selected and classified as transposable elements according to Repbase [43] for *E. oleifera* and *E. guineensis*, respectively*.* The complete databases are provided in Additional file 1 and a summary of their contents is presented in Additional file 2. The consensus sequences that were classified as either chimeric or potential host genes (272 and 278 sequences, respectively) were subsequently removed from TEdenovo’s output. Among the remaining 3475 Eo consensus sequences, 72 % are assigned to Class I and 19 % to Class II, whereas for Eg (9915 consensus sequences) the respective proportions are 39 and 8 %, respectively (Fig. 1). Remarkably, the main difference between the two sets of consensus sequences is the considerably larger proportion of unclassified repeats (*NoCat*) in *E. guineensis* (54, vs. 9 % for *E. oleifera).*

Among successfully classified repeats, the most represented groups in both genomes are, in decreasing order: RXX (unclassified retrotransposons) and potentially non-autonomous retrotransposons such as LARDs and TRIMs; 58 % for Eo; 60 % for Eg), DTX (transposons; 20 and 16 %, respectively) and RLX (LTR retrotransposons; 15 and 16 %, respectively) (Fig. 1).

**Fig. 2:**
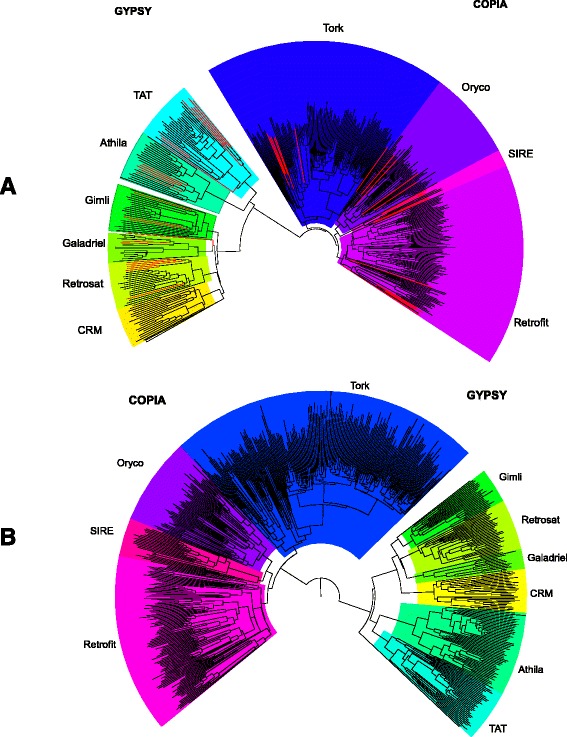
Phylogenetic analysis of LTR retrotransposons consensus sequences predicted from the *E. oleifera* (a) and *E. guineensis* (b) genome sequences. Phylogenetic trees were based on amino-acid alignments of the reverse transcriptase (RT) domains (see Methods for details) The classification was done according the RT reference domains (*red lines*) downloaded from GyDB [44]

The RLX consensus sequences were further classified into lineages and families [44]. Significant sequence similarities were detected for 337 and 491 RLX consensus sequences from Eo and Eg respectively. The analysis of the resulting Neighbor-Joining trees (Fig. 2) shows that most of the LTR retrotransposon lineages that have previously been identified in other Angiosperm genomes are represented in both *E. oleifera* (Eo) and *E. guineensis* (Eg). Interestingly, lineage diversity appears to be similar between both oil palm genomes. It is also worth noting that all the LTR retrotransposon consensus sequences (RLX) identified by TEdenovo in both *Elaeis* genomes were classified as incomplete elements.

### TE abundance in oil palm genomes (*E. oleifera* and *E. guineensis*)

**Fig. 3:**
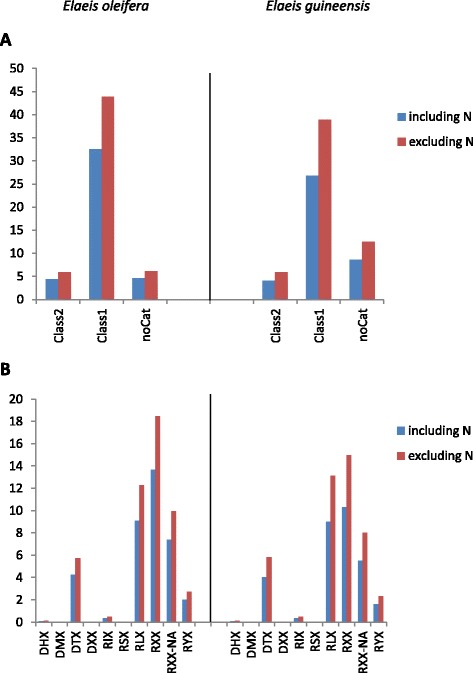
Percentage of identified TEs in the *E. oleifera* and in the *E. guineensis* genomic sequences. **a** Distribution of the elements among Class I, Class 2 and No Cat (Unclassified), **b** detailed distribution among the Repeat groups. The percentage of TEs is displayed as found in genomic sequences (in blue) including uncharacterized bases (N) and in genomic sequences excluding ‘N’ (in red)

The impact of TE populations on the genome sizes of *E. oleifera* (Eo) and *E. guineensis* (Eg) was estimated [45]. The TEdenovo output for Eo masks 41.39 % (580,386,071 bp) of available genomic sequences and 55.9 % when excluding unassigned nucleotides (N) from the analysis. Similar proportions are obtained for *E guineensis* assembly: 39.50 % (606,458,450 bp) and 68.8 %, respectively (Fig. 3). In the Eo genome, Class I and Class II consensus TEs mask 32.47 and 4.35 % of the genomic sequences, respectively, whereas the corresponding percentages are 26.8 and 4.08 % for Eg (Fig. 3a). In both genomes, the most abundant among identified TE categories are (in decreasing order): unclassified retrotransposons (RXX; Eo: 13.6 %; Eg: 10.3 %), LTR retrotransposons (RLX; Eo: 9.08 %; Eg: 9.02 %) and Non-autonomous LTR retrotransposons (RXX-NA; Eo: 7.35 %; Eg: 5.5 %) (Fig. 3b). Taken together, our results suggest that Class I elements form the majority of the TE component in both *E. oleifera* and *E. guineensis* genomes, with LTR retrotransposons constituting the largest subclass in both instances.

Among the RLX TE consensus sequences that were previously classified, we further studied the respective contribution of LTR retrotransposons lineages and families to the *E. oleifera* and *E. guineensis* genomes. In both *E. oleifera* and *E. guineensis* genomes, the frequency ratio of *Copia* to *Gypsy* retrotransposons is of ~5:1. According to our analyses, *Tork* is the most represented lineage in both genomes (Additional file 3) and *Sto-4* is largely predominant among families (Additional file 4).

### Characterization of full-length LTR retrotransposons in *E. guineensis*

Since LTR retrotransposons represent the major part of the TE fraction in the *E. guineensis* genomes, subsequent analyses were focused on this particular class of elements. The RLX consensus sequences previously retrieved from the TEdenovo analysis were found to be incomplete, and therefore we used the LTR_STRUC algorithm [46] in order to identify and localize the corresponding full-length copies in the genome of *E. guineenis*.

We collected a total of 583 full-length elements (see their sequences displayed in Additional file 5 and the summary of the full-length elements collection in Table 1). Among them, 241 (41.3 %) were assigned to the C*opia* superfamily (RLC) and 151 (25.9 %) to the G*ypsy* superfamily (RLG). The remaining 191 elements (32.7 %) could not be classified since no similarity to known Reverse Transcriptase, Integrase nor RNAseH coding domains could be found (Table 1). They were considered hereafter as putative non-autonomous retrotransposons (RXX-NA). However, an interesting point is that 105 of these non-assigned elements included either a putative GAG coding domain alone or both a GAG and a protease (PR) coding domains, suggesting than some of them might belong to the recently discovered subclass of TR-GAG elements [47].

**Table 1:** Structural characteristics of the full-length LTR retrotransposons of oil palm *E. guineensis*

Superfamily/lineage	Groups	Elements		LTR		Copies (70–70 %)	
		Number	Avg. length (bp) (min-max)	Avg. % identity	Avg. length (bp) (min-max)	Number	Genome coverage (%)
*Copia*							
*Oryco*	25	36	4796	91.85	338		
			4536–5627		240–469		
*Sire*	8	8	9063	89.34	1170		
			4538–11,043		347–1475		
*Retrofit*	74	97	5075	91.62	269		
			2198–9109		83–414		
*Tork*	66	73	7539	88.43	1069		
			4118–10,744		131–248		
Undefined clade	26	27	8108	89.21	1473		
			1408–10,381		168–2668		
Subtotal	199	241	6252	90.34	863	4816	2.32
			1408–11,043		83–2668		
*Gypsy*							
*Athila*	10	10	10,438	91.07	1411		
			1171–9780		875–1697		
*Tat*	58	84	10,078	90.64	666		
			5572–11,478		350–1129		
*CRM*	16	20	6455	89.51	712		
			8463–5084		296–1380		
*Del*	3	3	8624	86.07	1454		
			6730–10,443		305–2468		
*Galadriel*	3	3	7463	90.83	1777		
			5826–10,130		533–3861		
*G-Rhodo*	1	1	5506	90.40	448		
					447–450		
*Reina*	15	16	5921	91.62	607		
			5085–10,286		266–2490		
Undefined clade	13	14	6561	88.07	824		
			2113–11,900		110–1413		
Subtotal	119	151	8744	90.30	987	1934	1.06
			2113–11,900		110–3861		
Putative non autonomous (RXX-NA)	174	191	4911	88.20	828	3804	1.01
			1279–11,793		83–5259		
Total	492	583	6458	89.63	753	10,554	4.39
			1279–11,900		83–5259		

As previously, RLC and RLG elements were further classified into lineages. The results obtained with the full-length LTR retrotransposon elements were essentially identical to those previously described with the partial TE consensus sequences (Additional file 6).

Further examination of the internal ORFs revealed that the majority (83 %) of the full-length RLC and RLG elements encode either four (108 elements) or five (215 elements) protein coding domains (Additional file 7A). In accordance with current standards of TE classification [1], the elements belonging to the latter category (which represent 55 % of total full-length LTR retrotransposons) contain both the POL and the GAG coding regions that are required for transposition. They are therefore susceptible to include autonomous elements, however further sequence analyses show that these domains are most likely non-functional due to frameshifts and mutations. Also, we observe that the frequency of each protein coding domain is similar between both retrotransposons super-families, and that over 90 % of the elements include all three RT, INT and RH coding domains regardless of the superfamily (Additional file 7B). By contrast, LTR length is extremely variable, ranging from 83 to 5259 bp with an average value of 753 bp (Table 1). Nevertheless, within most lineages the average LTR length of oil palm retrotransposons is in agreement with data collected from other plant species.

### Full-length LTR retrotransposon copy number and chromosomal distribution

In order to cluster the 583 full-length LTR retrotransposons of oil palm into families based on sequence relatedness, we eliminated sequence redundancy according to the recommendations of Wicker et al. [1], i.e. elements are deemed related if a sequence identity of at least 80 % is detected across 80 % of the length of the retrotransposon. However, due to its high level of stringency and, possibly, to the structural diversity of LTR retrotransposons in the oil palm genome, this analysis was unable to detect related elements in our case (results not shown). An empirically determined threshold of 70 % (of sequence identity)—70 % (of sequence length) was finally used and enabled the identification of 492 groups, including 199 *Copia*, 119 *Gypsy* and 174 putative non-autonomous retrotransposons (RXX-NA), as potential TE families (Table 1). Most of these groups include a single element, further strengthening the hypothesis of a high level of sequence diversity of LTR retrotransposons in oil palm. However, our analysis is based on the current release of the *E. guineenis* genome [39] and an improvement of both the overall quality of genomic sequences and scaffold size of the assembly are necessary before this assumption can be confirmed.

The number of full-length LTR retrotransposon copies in the *E. guineenis* genome was estimated using one reference element from each of the 492 groups. However, because of the high level of sequence diversity among these elements, it was not possible to assign each copy to a single reference sequence unequivocally, and as a result copy number was determined globally for each superfamily.

A total of 10,554 full-length copies were detected, representing 4.39 % of the oil palm genome assembly (Table 1). Copies belonging to the *Copia* superfamily (4816) displayed the highest rate of genome coverage (approximately 2.32 %), whereas the *Gypsy* superfamily (1934 copies) accounted for 1.06 %.

**Fig. 4:**
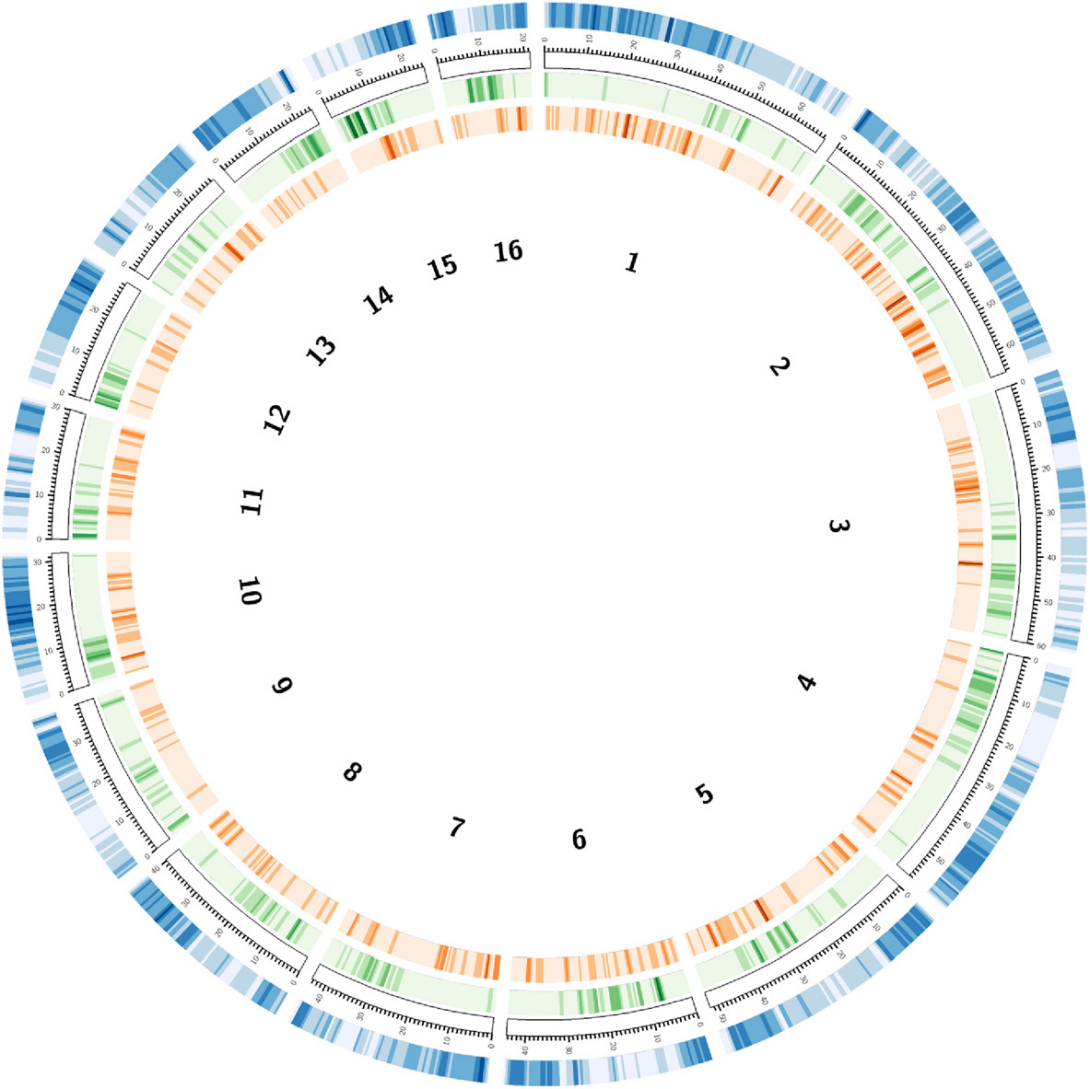
Chromosomal distribution full-length LTR retrotransposons in the *Elaeis guineensis* genome. Green track: *Copia* elements; orange track: *Gypsy* elements; blue track: predicted genes. The intensity of the coloring is directly proportional to the respective sequence densities

To gain insight into possible correlations between the respective distribution of predicted coding sequences and full-length LTR retrotransposons, we plotted the LTR retrotransposon density along the 16 *E. guineensis* pseudo-chromosome sequences accounting for 43 % of the whole genome assembly [39]. Among the 10,554 full-length LTR retrotransposon copies identified previously, 5703 (~54 %) could be mapped to the 16 assembled pseudo-chromosomes (Fig. 4). The average LTR retrotransposon density was 8.75 sequences per Mb with 3.43 and 1.96 respectively for *Copia* and *Gypsy*. In addition, the analysis of TE distribution with respect to predicted genes showed that full-length *Gypsy* elements were distributed uniformly across the 16 pseudo-chromosomes, irrespective of gene location, whereas a highly significant negative correlation was observed between the density of full-length *Copia* elements and gene density (*R* = −0.46, *P* = 0.0000) (Additional file 8). This latter result seems to indicate a higher abundance of *Copia* in gene-poor regions compared to gene-rich regions in the *E. guineensis* genome.

### Transcriptional activity of full-length LTR retrotransposons

**Fig. 5:**
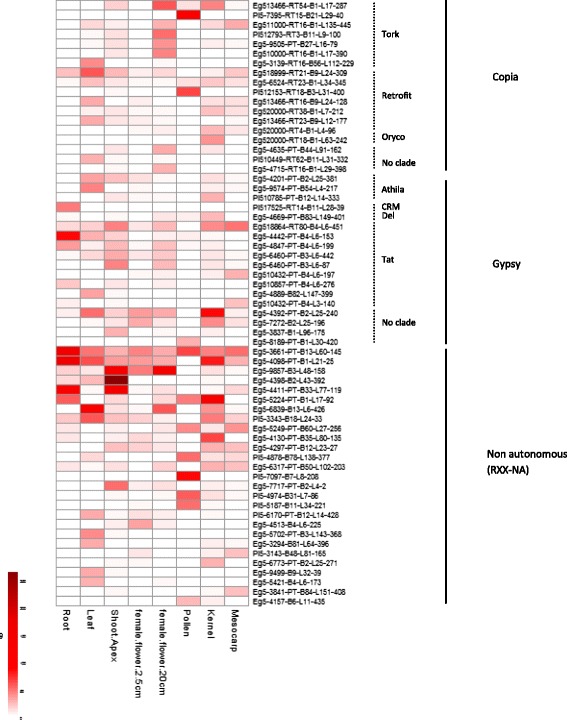
Expression map of full-length LTR retrotransposons in *Elaeis guineensis* tissues. The intensity of the coloring is directly proportional to the relative expression of the retrotransposons (see text for details)

The relative expression of the 583 full-length LTR retrotransposons was assessed through the computational analysis of publicly available RNAseq libraries from eight different oil palm tissues (see Methods). Overall, most of the elements show a low level of transcription regardless of the tissue, whereas 63 retrotransposons are expressed in at least one of the studied tissues. Among these, most are transcribed in a limited number of tissues with no significant clustering (Fig. 5). It is interesting to note that elements classified as potential non-autonomous LTR retrotransposons (RXX-NA, Table 1) account for 57.8 % of the overall transcriptional activity related to these 63 LTR retrotransposons in the eight tissues studied, *vs.* 19.3 % and 22.8 % for *Copia* and *Gypsy* elements, respectively (Additional file 9A). Moreover, when comparing expression levels related to these 63 elements between tissues, we observe that it is the highest in shoot apices (18.2 % of the cumulated expression of these elements in all eight libraries, Additional file 9B) and the lowest in young female flowers (6.3 %). Intriguingly, this share is increased to 13.4 % in more mature female flowers, mostly due to the upregulation of *Tork* elements and, to a lesser extent, that of *Tat* elements which translate into 62- and 4-fold increases in count numbers fort these lineages between both inflorescence stages, respectively. Only one element (Eg5-3661-PT-B13-L60-145; RXX-NA) shows transcriptional activity in all tested tissues, whereas the Eg5-4398-B2-L43-392 (RXX-NA) element generates the strongest expression signal observed in this study, in the shoot apex.

### Putative insertion time and evolution of LTR retrotransposon populations in the *E. guineensis* genome

In order to infer the evolutionary history of LTR retrotransposon populations in the oil palm genome, we evaluated sequence divergence between the 5’ and 3’ LTRs of each full-length element. Because of the requirements of the transposition mechanism, both LTR sequences of a single retrotransposon are 100 % identical at the time of its insertion into the genome. Through time, they progressively diverge from one another by accumulating mutations, such as nucleotide substitutions. It is therefore possible to calculate the nucleotide substitution rate between both LTRs in order to roughly discriminate the respective insertion times of different retrotransposon populations [48].

**Fig. 6:**
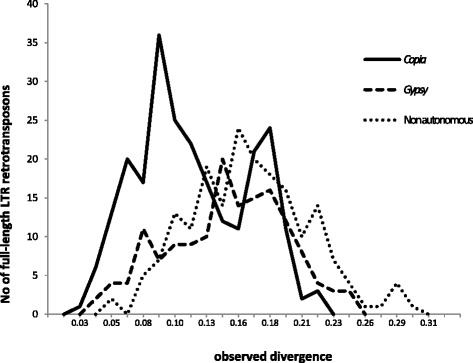
Estimated insertion of full-length LTR retrotransposons into the oil *E. guineensis* genome. The horizontal axis represents sequence divergence; see text for details

Our results, illustrated in Fig. 6, suggest that the oil palm genome underwent several waves of LTR retrotransposon amplification events, with different temporal patterns of transpositional activity for the main superfamilies *Copia* and *Gypsy*. Massive insertion events of *Copia* retrotransposons into the oil palm genome were displayed in two distinct peaks. By contrast, we observe a single peak for the insertion of *Gypsy* elements and Non-Autonomous LTR retrotransposons. Finally, we detect very few recent insertion events in our dataset, with the notable exception of the Pi519857_RT4_B11_L9_394 element (*Copia* superfamily, *Oryco* lineage).

## Discussion

In the original paper describing both oil palm genomes [39], the repeat content of *E. guineensis* had been estimated to approximately 57 % of its 1.8 Gb genome with a large prevalence of LTR retrotransposons, while no such analysis had been performed in *E. oleifera*. Thus, to our knowledge the present study constitutes the first attempt to compare TE populations between both oil palm genomes and to further characterize LTR retrotransposon families and lineages in the major oil crop *E. guineensis*. In other plant species, genome-wide analyses of TE populations have not only provided clues on the individual history of each genome but they have also yielded useful information about the origins of modern-days crop genomes through domestication, speciation and hybridization. In wheat, such studies have demonstrated the occurrence of differential dynamics of TE proliferation between the A and B sub-genomes, both prior and after allotetraploidization [49]. In rice, the comparison of the transpositional history of LTR retrotransposons between the Japonica and Indica sub-species has provided evidence for two independent domestication processes in Asian rice [50].

As a first step in our study, we have annotated the transposable elements from both *Elaeis* genomes through a *de novo* approach [42]. While we found that the overall repeat content is nearly identical between both genomes, the comparison between the two TE databases revealed the occurrence of 6 times more Unclassified Repeats in the *E guineensis* genome compared to *E. oleifera*. This discrepancy in the distribution of TE categories is most likely due to differences in both sequencing completion and assembly quality between both draft genomes, resulting in a lesser coverage of this TE subclass in *E. oleifera*. However, the distribution of all other TE categories was similar between the *E. guineensis* and *E. oleifera* genomes, with a large prevalence of Class I elements. Furthermore, among the LTR retrotransposons that form the major part of the TE fraction, we discovered a comparable diversity within the *Gypsy* and *Copia* super-families between both oil palm genomes. Our results also highlighted a remarkable expansion of the same retrotransposon lineages (namely *Retrofit* and *Tork*), indicating that they were intensively active during the evolution of both palm genomes. In both species, *Copia* elements are more abundant than *Gypsy* and one LTR retrotransposon family, named *Sto-4* and belonging to the *Copia* superfamily, is the most represented. This result is consistent with a previous analysis of 32 Reverse Transcriptase coding sequences, from which it was extrapolated that *Copia* elements might make up about 6 % of the *E. guineensis* genome [37]. Also, our findings are in accordance with those from Singh and co-workers [39] since they observed a significant expansion of a RIRE-like member of the *Sto-4* family in the *E. guineensis* genome. An interesting point is that the *RIRE1* element has originally been identified in *O. australiensis*, a wild relative of rice [51], where its 30,000 copies represent 27 % of the host genome. The extreme similarity in LTR retrotransposons distribution between both *Elaeis* genomes makes it tempting to hypothesize that the massive amplification of some families, especially those belonging to the *Tork* lineage, occurred before both palm genomes diverged from each other. According to some authors, this divergence took place 51 Mya ago [39] but other studies using different datasets and methods suggested that the speciation event might be much more recent: between 7 Mya ago [52] and 15 Mya ago according to Baker and Couvreur [53]. Clearly, more in-depth phylogenetic analyses of the oil palm genomes are needed to ascertain both the time frame of this event and the dating of TE insertions since the genomic organization of TE populations results from a dynamic balance between amplification bursts and recombination events leading to DNA loss [1, 6, 54, 55].

It must be kept in mind that the approach that were used here, and which are based on the *de novo* reconstruction of TE consensus sequences from available genome contig sequences, may have led to an underestimation of the repeat content. In any case, the present study represents a first step towards the construction of a comprehensive oil palm TE catalogue.

Since LTR retrotransposons represent the vast majority of the Transposable Elements found in oil palm genomes, we have further characterized full-length LTR retrotransposons in the *E. guineensis* genome. Interestingly, when analyzing retrotransposon density across the genome, we find a preferential insertion of full-length *Copia* elements in relatively gene-poor regions of the assembled pseudo-chromosomes, whereas the *Gypsy* elements appear to be randomly distributed. In oil palm, previous *in situ* hybridization experiments performed by Castilho et al. [36] led to a similar conclusion about the *Copia* elements, and Schnable et al. [3] also observed such a differential distribution of both super-families in maize. Comparisons performed between partially or completely sequenced plant genomes have shown that LTR elements are mostly concentrated in gene-poor regions, with variations according to superfamily or lineage [56]. In relatively gene-rich regions however, the dispersion of LTR retrotransposons appears to be greater within small plant genomes (<500 Mb) as opposed to large ones, where they occur as stretches of nested elements [56].

We find that, although most of the elements identified in the *E. guineensis* genome include the protein coding domains that are required for transposition, the sequences are disrupted by premature stop codons or frameshifts, indicating that they might no longer be functional. Furthermore, the analysis of the sister LTRs from each of these retrotransposons failed to detect any element displaying 100 % nucleotide identity between these repeats, which is a hallmark of recently inserted TEs. Taken together with estimates of LTR sequence divergence and the high degree of structural diversification observed between retrotransposon lineages, this result suggests that no significant insertion event occurred recently in the African oil palm genome. However, in the absence of data on base substitution rates in oil palm genes and TEs, it is not possible to improve on the dating of these insertion events through the use of a “molecular clock” such as was used in the rice genome [57]. Despite this limitation, our analysis shows that the full-length *Copia* and *Gypsy* elements studied were inserted into the oil palm genome around the same period of time but exhibited distinct dynamics. Interestingly, no recent insertion event was inferred, further strengthening the hypothesis that the LTR retrotransposons of the oil palm genome are most likely transpositionally inactive. This conclusion is in contrast to observations made in other plant genomes such as maize [58], rice [55, 57] and coffee [59], in which recent retrotransposon insertion events have been detected but quite similar to the situation in the banana [60] and olive tree genomes [61]. In the latter genome, transpositional activity from LTR retrotransposons has been shown to be decreasing over time but active copies are nevertheless still detectable.

These various clues to the lack of transpositional activity from the LTR retrotransposons analyzed, as well as the high proportion of retrotransposons classified as non-autonomous in our study such as LARD [62] and TRIM [63], paint a picture of the *E. guineensis* genome as a landscape where TEs are mostly, if not completely, immobile. However, the presence in the genome of trancriptionally and/or transpositionally active copies, enabling the *trans*-complementation of these presumably inactive elements, cannot be excluded at this stage. Indeed, our investigation of the transcriptional activity of our full-length LTR retrotransposons shows that some of them are expressed, albeit mostly at low levels, in different oil palm tissues even though their transcriptional and/or their transpositional autonomy is most likely impaired because of the accumulated mutations. Our analysis also shows that the expression levels of these transcriptionally active retrotransposons are highly variable between both lineages and tissues. We hypothesize that this activity could be related to the Developmental Relaxation of TE silencing (DRTS) [64] that has been shown to result in relatively elevated TE expression, notably in shoot and inflorescence meristems of both maize and rice [65–67]. Although the exact role of this phenomenon is yet to be elucidated, it has been proposed to contribute to the reinforcement of small RNAs-mediated TE silencing through their transient derepression in specific tissues, as well as to the epigenetic regulation of both genes and genome in connection with cell specification and plant development [64]. Overall, these data suggest that these elements could still have the potential to interfere with the expression of neighboring genes, through either the production of read-through or antisense transcripts [10] or the alteration of epigenetic marks [68, 69], and lead to phenotypic variations. Our group has recently demonstrated that the splicing of the *EgDEF1* gene, which is believed to be involved in the *mantled* floral phenotype, is strongly affected in variant flowers, possibly as a consequence of the intronic insertion of an inactive *Gypsy* retrotransposon [70]. Additional work will be required to further explore the interactions between the mechanisms regulating genes and TE expression in the oil palm genome.

## Conclusions

The present study presents the most comprehensive description of oil palm LTR retrotransposons to date. Our results, which reveal a congruence in the tranpositional history of LTR retrotransposons between *E. oleifera* and *E. guineensis*, will provide crucial information for dating their divergence and further, to elucidate the history of genome evolution in the Arecaceae palm family. Moreover, our TE database will be a helpful resource in future studies aiming at assessing the possible contribution of LTR retrotransposons to genome and transcriptome variations resulting from the *in vitro* somatic embryogenesis process, especially in the context of the oil palm *mantled* phenotype.

## Methods

### Data source

The oil palm *Elaeis guineensis* and *Elaeis oleifera* genome sequences generated by Malaysian Oil Palm Genome Programme (MyOPGP) [39] have been downloaded from the NCBI and MPOB web sites (http://www.ncbi.nlm.nih.gov/assembly/GCA_000441515.1/; http://genomsawit.mpob.gov.my/genomsawit/, in August 2013; P5-build and EG5-linked assemblies for *E. guineensis* and O8-build for *E. oleifera*).

### Annotation of transposable elements

A combination of manual approaches and automated programs (REPET package V.2.2-RC; [42]) were used to identify, classify and annotate repeated sequences from the largest scaffolds (size > 300 kbp) assembled for each of the studied genomes. The sequences that were investigated include 991 scaffolds amounting to a total of 730,618,412 bp of genome sequence from the O8-build and 846 scaffolds representing 1,068,102,326 bp from the P5-build, respectively. TE consensus nucleotide sequences were classified according to the Repbase database [43] and named according to the classification proposed by Wicker et al. [1]: DHX (Helitron), DMX (Maverick), DTX (TIR Transposon), DXX (MITE) for Class II elements, and RIX (LINE), RLX (LTR Retrotransposon), RSX (SINE), RXX (unclassified or non-autonomous retrotransposons), RYX (DIRS) for Class I element. Consensus sequences assigned as LTR retrotransposons were further classified through the phylogenetic analysis of their reverse transcriptase (RT) amino-acid domains: putative RT coding domains were first identified in consensus nucleotide sequences using BLASTX [71] and translated using Genewise [72], then the resulting RT amino acid sequences (with a minimum length of 150 residues) and reference RTs from Gypsy Database 2.0 [44] were aligned with ClustalW to construct a NJ tree that was finally edited with FigTree (http://tree.bio.ed.ac.uk/software/figtree/). Repeatmasker [45] was used with default parameters so that sequences with less than 80 % identity to the reference sequence were masked.

The LTR_STRUC 1.1 algorithm [46] was used with default parameters in order to detect full-length LTR retrotransposons among the complete dataset of *Elaeis guineensis* scaffolds (1,535,150,282 bp). The following structural definition was used for the full-length LTR retrotransposon, regardless of its transcriptional or transpositional ability: a repeat element that i) is delimited by highly similar 5’ and 3’ LTRs; ii) has generated a Target Site Duplication (TSD) on each border of the insertion site into the host genome; iii) includes putative primer binding site (PBS) and polypurine tract (PPT) sequences at the 5’ and 3’ the of its internal sequence, respectively.

### Annotation and phylogenetic analysis of full-length LTR retrotransposons

Raw results from the LTR_STRUC detection were analyzed as follow to construct an oil palm full-length LTR retrotransposons reference library. This data was classified into *Gypsy* (RLG) and *Copia* (RLC) superfamilies according to their similarity with the content of the GyDB domain libraries [44] and the occurrence and respective location of the protein coding domains for the Reverse-transcriptase (RT), Integrase (INT) and RNAseH (BLASTX E-value cut-off: 1e-4). Sequences were classified into the RXX category if only the GAG (capsid) and PR (protease) coding domains, or the GAG alone, were detected, or if no sequence similarity could be found. Predicted sequences larger than 12 kb were not retained for further analysis. Classification of predicted RLG and RLC full-length LTR retrotransposons was confirmed and completed by phylogenetic analyses as described previously, using four previously published oil palm RT domains (GenBank accessions AJ507412 to AJ507415 [38]).

### Copy number estimation, distribution and insertion time

All identified full-length LTR retrotransposons were clustered into families or group using the CD-HIT software [73] with a minimum of 70 % of nucleotide identity and a minimum sequence coverage of 70 % between related elements. Within each family, the longest sequence displaying a high percentage of nucleotide sequence identity between both LTR regions was selected as the reference sequence. The copy number of each super-family was determined using Censor [74]. A copy is considered as complete if it covers a minimum of 70 % of the reference sequence with a minimum of 70 % of nucleotide identity. The density of retrotransposon distribution along pseudo-chromosomes was calculated using a home-made shell script, with a 1 Mbp sliding window (step size of 500 kbp) and plotted using CIRCOS [75].

The insertion times of the previously identified full-length LTR retrotransposons were estimated based on the sequence divergence between the 5’ and 3’ LTR of each element, as determined through successively aligning the sequences using Stretcher then implementing the Kimura 2-parameter method in Distmat (EMBOSS package). An average base substitution rate of 1.3E-8 was used in accordance with Ma and Bennetzen [57].

### Transcriptional analysis of LTR retrotransposons

The transcriptional analysis was carried out using data deposited into NCBI’s databases (Bioproject number PRJNA201497). Eight sets of oil palm (*Elaeis guineensis*) transcriptome data from different tissues were re-analyzed: root (SRX278062), leaf (SRX278048), shoot apex (SRX278055), young female flower (SRX278052), mature female flower (SRX278053), pollen (SRX278051), kernel (SRX278018) and mesocarp (SRX278017). Data quality was evaluated with FastQC [76] and low quality reads were excluded with Cutadapt [77]. Reads were mapped against our full-length LTR retrotransposons reference library using the BWA-MEM package with default parameters [78]. Samtools [79] was used to calculate the number of mapped reads (counts) for each reference sequence and normalization was performed using the EdgeR package [80]. The graphical representation of full-length LTR-retrotransposons expression in the different tissues was generated by the pheatmap R package [81].

### Availability of data and materilas

The data sets supporting the results of this article are included within the article and its additional files.

## Additional files

Additional file 1:
***De novo*** **consensus TE databases.** Contains the *E. oleifera* (E08_denovoLibTEs.txt) and *E. guineensis* (p5_denovoLibTEs.txt) databases. (ZIP 15,038 kb) Additional file 2:
**Summary of the contents of the** ***E. oleifera*** **and** ***E. guineensis*** **TE databases.** (PDF 9 kb) Additional file 3:
**Sequence coverage of LTR retrotransposon lineages in the** ***E. oleifera*** **(Eo) and in** ***E. guineensis*** **(Eg) genomes. **(PDF 35 kb) Additional file 4:
**Sequence coverage of LTR retrotransposon families in the** ***E. oleifera*** **(Eo) and in** ***E. guineensis*** **(Eg) genomes.** (PDF 356 kb) Additional file 5:
**Overview of LTR_STRUC results.** For each full-length LTR retrotransposon, superfamily, putative lineage, overall length, identity percentage between both LTR sequences, target site duplication (TSD), polypurine tract (PPT) and tRNA binding site are provided, as well as the sequences of the element and of its LTRs. (XLSX 1543 kb) Additional file 6:
**Phylogenetic analysis of full-length LTR retrotransposon sequences predicted from the** ***E. guineensis*** **genome.** See Methods for details. (PDF 332 kb) Additional file 7:
**Protein coding domain composition of full-length LTR retrotransposons of oil palm** ***E. guineensis*** **.** A: Number of protein coding domain identified per retrotransposon (figures correspond to the number of elements displaying either 1, 2, 3, 4 or 5 domains). B: Frequency of the different protein coding domain in the *Copia* and *Gypsy* superfamilies. AP = protease, INT = integrase, RT = reverse transcriptase, RH = ribonuclease, GAG = capsid. (PDF 174 kb) Additional file 8:
**Relationship between the density (sequences per Mb) across chromosomes of predicted coding sequences and that of** ***Copia*** **(A) and** ***Gypsy*** **(B) full-length elements.** (PDF 153 kb) Additional file 9:
**Transcriptional activity of 63 LTR retrotransposons.** A: Percentage of normalized read counts per superfamily. B: Percentage of normalized read counts per tissue. (PDF 178 kb)

## Abbreviations

DIRS: Dictyostelium intermediate repeat sequence
LARD: Large retrotransposon derivative
LINE: Long interspersed nuclear element
LTR: Long terminal repeat
MITE: Miniature inverted-repeat transposable element
SINE: Short interspersed nuclear element
TIR: Terminal inverted repeat
TRIM: Terminal-repeat retrotransposon in miniature
