# Supplementary material for: Genome-wide analysis of LTR-retrotransposons in oil palm
Source: BMC Genomics. 2015 Oct 15;16:795. doi: 10.1186/s12864-015-2023-1 (PMC4608283; doi:10.1186/s12864-015-2023-1)
Supplement: Additional file 2: — Summary of the contents of the E. oleifera and E. guineensis TE databases. (PDF 9 kb) [file 12864_2015_2023_MOESM2_ESM.pdf]

	E. oleifera	*E. guineensis*
**Number of genomic scaffolds > 300 kb**	991	846
**Cummulative length of genomic scaffolds > 300 kb**	730,618,412	1,068,102,326
**Number of TE consensus found by REPET**	3,475	9,915
**Number of annotated TE consensus found by REPET:**		
**Class I**	**2,493**	**3,82**
ClassII RIX	88	138
ClassII RLX	475	720
ClassII RSX	23	79
ClassII RXX	1,812	2,754
ClassII RYX	95	129
**Class II**	**658**	**763**
ClassI DHX	12	22
ClassI DMX	5	2
ClassI DTX	635	730
ClassI DXX	6	9
**NoCat (unclassified)**	**324**	**5,332**
