# Supplementary figures and images for: Genome-wide analysis of LTR-retrotransposons in oil palm
Source: BMC Genomics. 2015 Oct 15;16:795. doi: 10.1186/s12864-015-2023-1 (PMC4608283; doi:10.1186/s12864-015-2023-1)

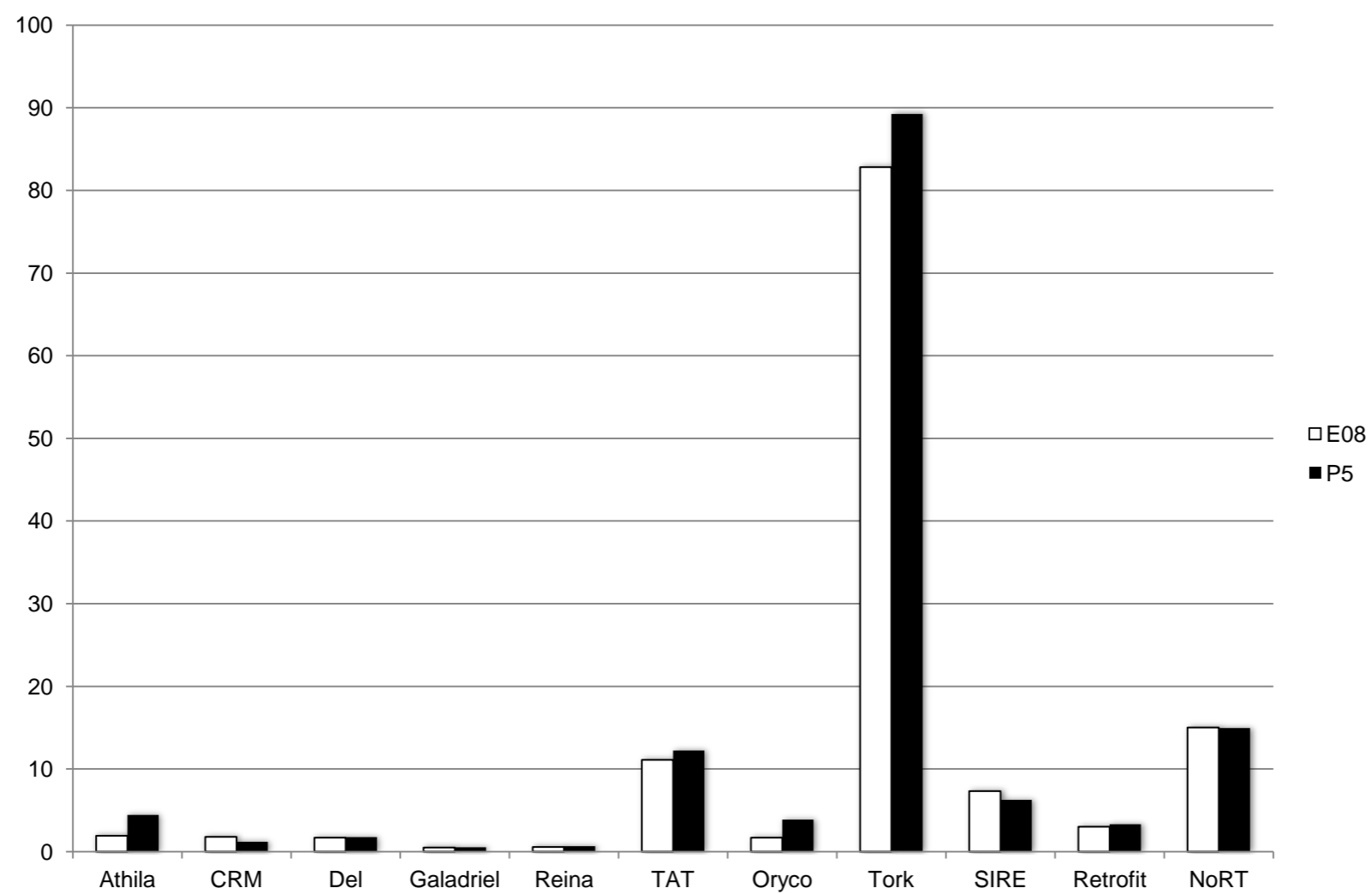

Supplement: Additional file 3: — Sequence coverage of LTR retrotransposon lineages in the E. oleifera (Eo) and in E. guineensis (Eg) genomes. (PDF 35 kb) [file 12864_2015_2023_MOESM3_ESM.pdf]

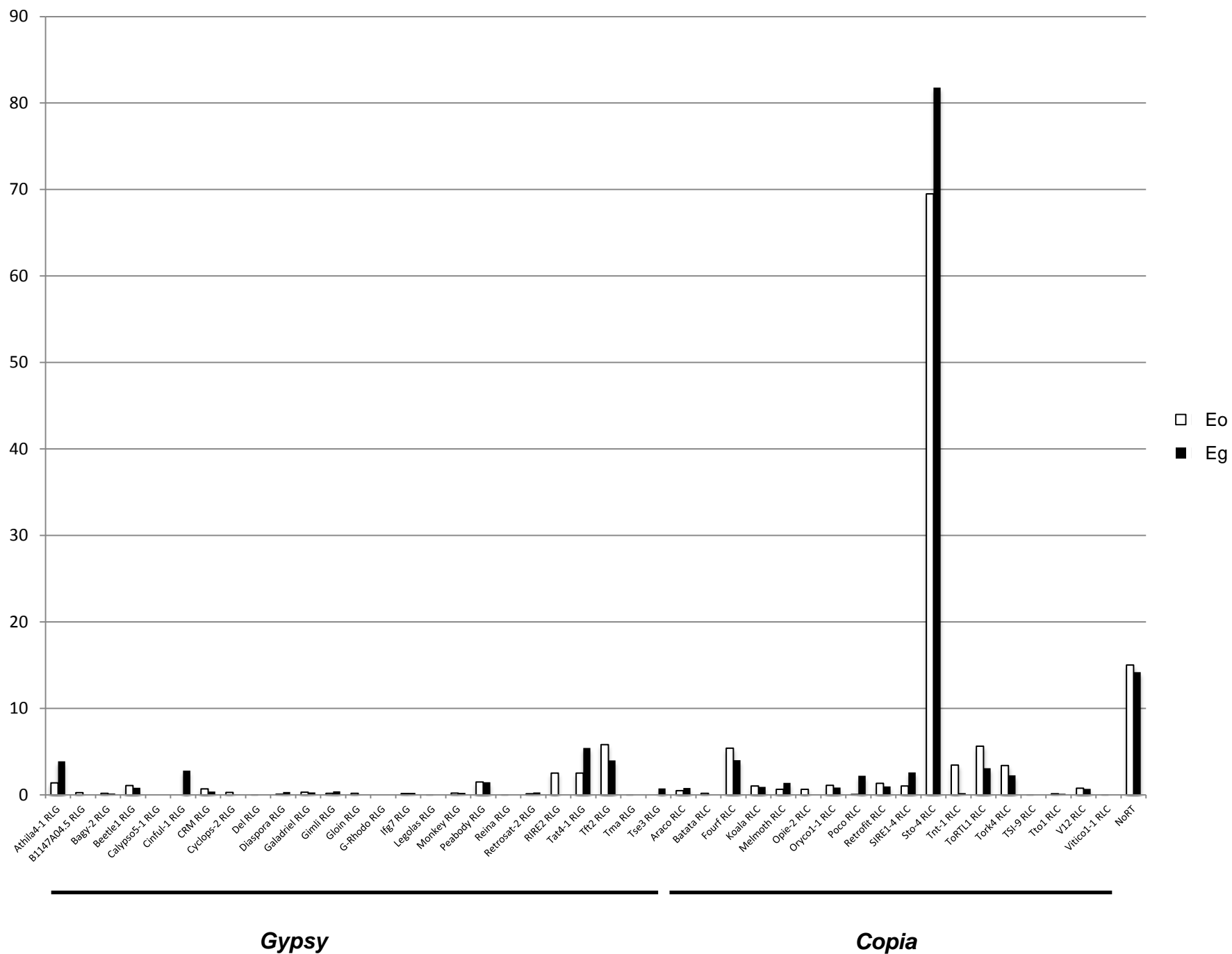

Supplement: Additional file 4: — Sequence coverage of LTR retrotransposon families in the E. oleifera (Eo) and in E. guineensis (Eg) genomes. (PDF 356 kb) [file 12864_2015_2023_MOESM4_ESM.pdf]

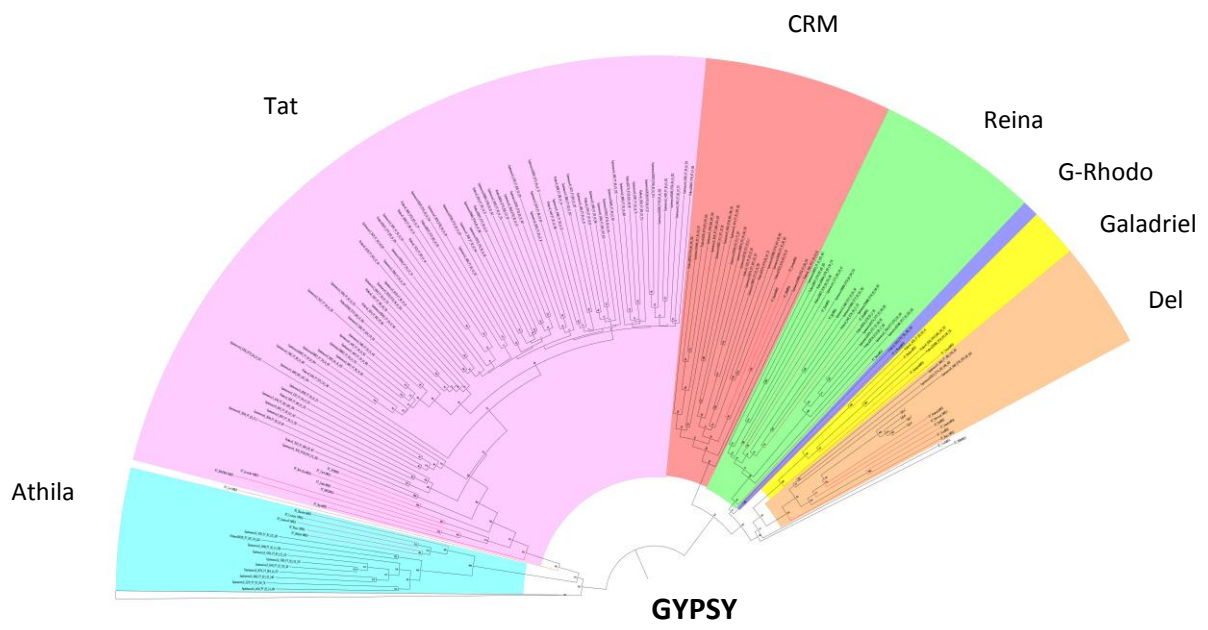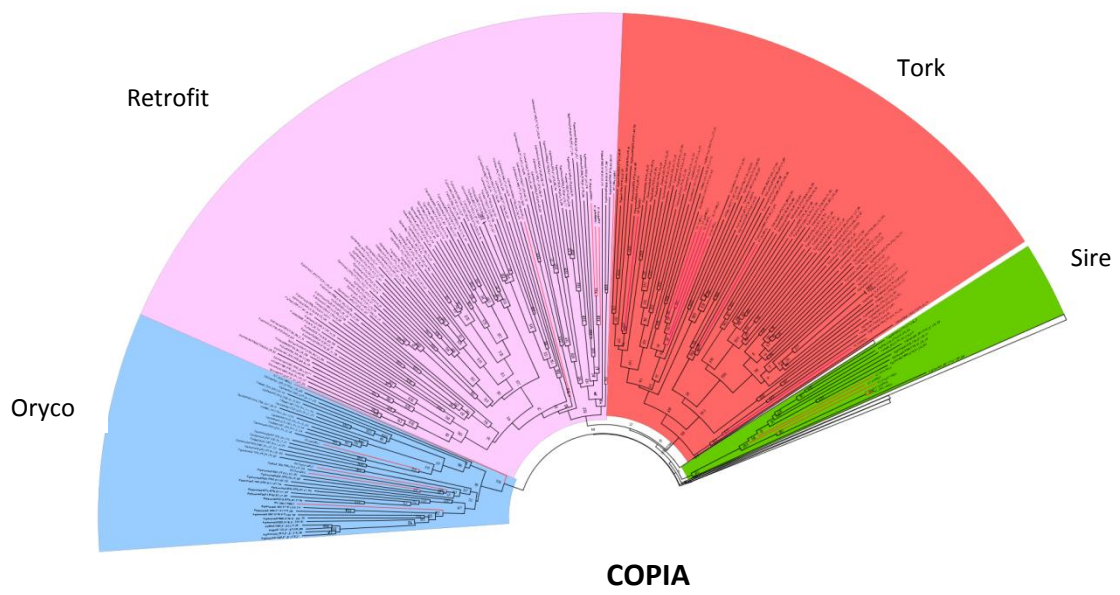

Supplement: Additional file 6: — Phylogenetic analysis of full-length LTR retrotransposon sequences predicted from the E. guineensis genome. See Methods for details. (PDF 332 kb) [file 12864_2015_2023_MOESM6_ESM.pdf]

**A**

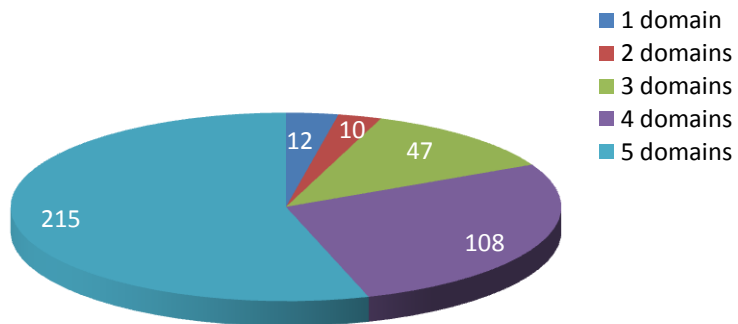

**B**

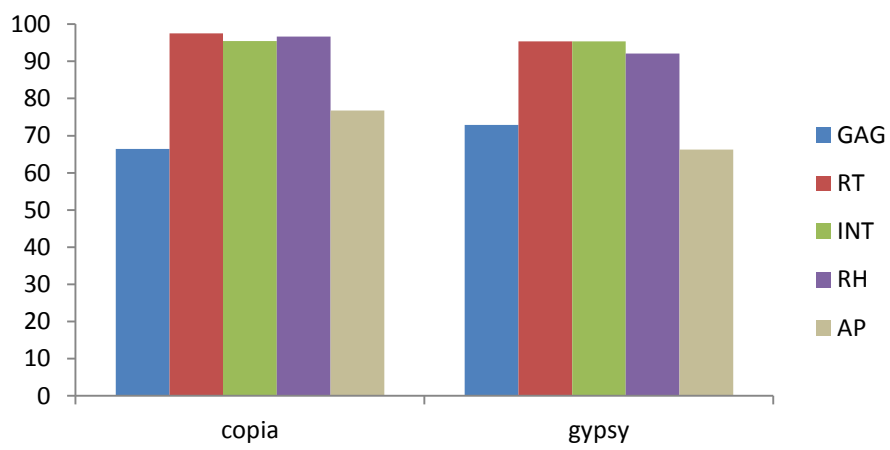

Supplement: Additional file 7: — Protein coding domain composition of full-length LTR retrotransposons of oil palm E. guineensis . A: Number of protein coding domain identified per retrotransposon (figures correspond to the number of elements displaying either 1, 2, 3, 4 or 5 domains). B: Frequency of the different protein coding domain in the Copia and Gypsy superfamilies. AP = protease, INT = integrase, RT = reverse transcriptase, RH = ribonuclease, GAG = capsid. (PDF 174 kb) [file 12864_2015_2023_MOESM7_ESM.pdf]

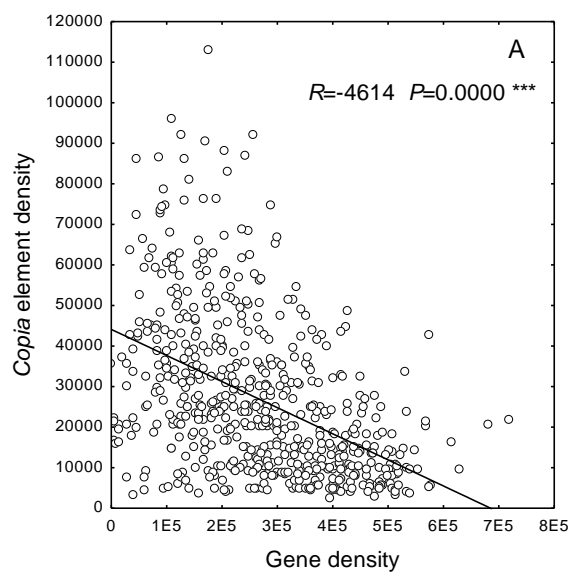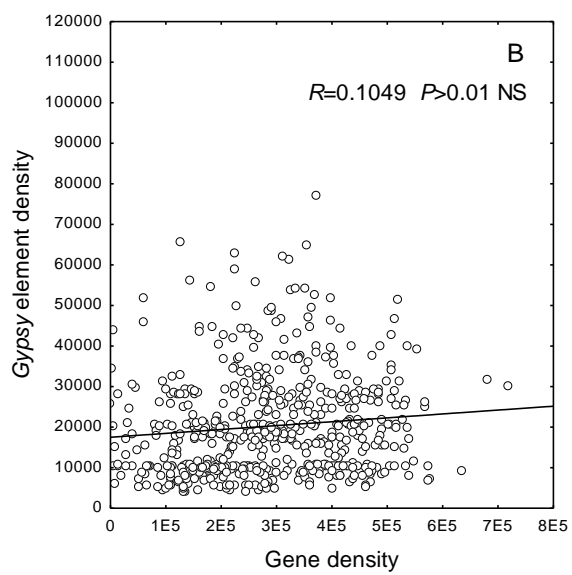

Supplement: Additional file 8: — Relationship between the density (sequences per Mb) across chromosomes of predicted coding sequences and that of Copia (A) and Gypsy (B) full-length elements. (PDF 153 kb) [file 12864_2015_2023_MOESM8_ESM.pdf]

**A**

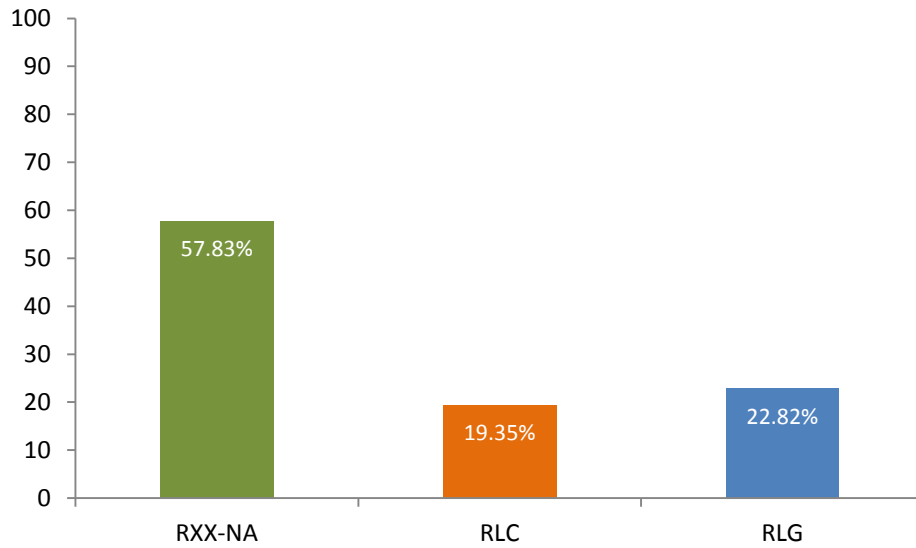

**B**

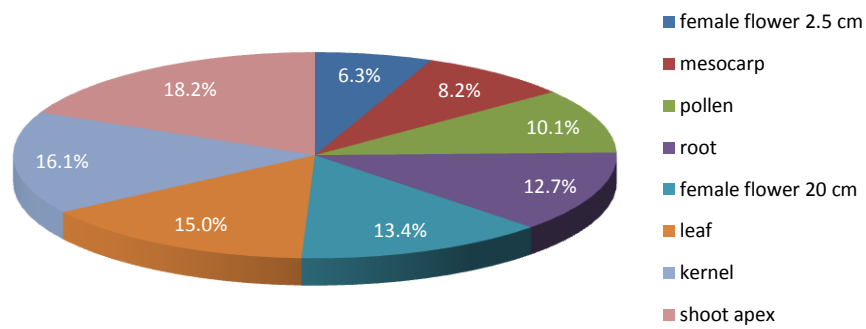

Supplement: Additional file 9: — Transcriptional activity of 63 LTR retrotransposons. A: Percentage of normalized read counts per superfamily. B: Percentage of normalized read counts per tissue. (PDF 178 kb) [file 12864_2015_2023_MOESM9_ESM.pdf]
